# Effects of Aquatic Exercise and Land-Based Exercise on Cardiorespiratory Fitness, Motor Function, Balance, and Functional Independence in Stroke Patients—A Meta-Analysis of Randomized Controlled Trials

**DOI:** 10.3390/brainsci11081097

**Published:** 2021-08-20

**Authors:** Daxin Li, Ping Chen

**Affiliations:** Department of Physical Education, Laoshan Campus, Ocean University of China, 238 Song Ling Rd, Qingdao 266100, China; lidaxin@stu.ouc.edu.cn

**Keywords:** aquatic exercise, land-based exercise, stroke, meta-analysis

## Abstract

The aim of this study was to evaluate the efficacy of aquatic exercise (AE) and land-based exercise (LE) on cardiorespiratory fitness, motor function, balance, and functional independence in stroke patients. Design: Through searching PubMed, Embase, Cochrane Library, Web of Science, CNKI, VIP and Wanfang Database, only randomized controlled trials (RCTs) were collected to study the effects of AE and LE on cardiorespiratory fitness, motor function, balance, and functional independence in patients with stroke. The included studies were evaluated for methodological quality by the Cochrane bias risk assessment tool, and statistical analysis was carried out by the Review Manage 5.3 and Stata 15.1 software. Results: The RCTs were collected between the earliest available date and April 2021. Eleven RCTs were included, including five studies with low risk and six studies with moderate risk. The total sample size used in the study was 369, which included 187 patients undertaking AE and 182 patients undertaking LE. The results of the meta-analysis showed that AE can significantly improve patients’ Berg Balance Scale (BBS) (MD = 5.19, 95% CI: 2.66 to 7.71, *p* < 0.0001), peak oxygen uptake (VO_2_peak) (MD = 3.49, 95% CI: 0.17 to 6.8, *p* = 0.04), Fugl–Meyer Assessment (FMA) (MD = 3.84, 95% CI: 1.64 to 6.04, *p* = 0.0006), and Functional Independence Measure (FIM) (MD = 6.1, 95% CI: 4.05 to 8.15, *p* < 0.00001). However, there was no statistically significant difference between the two exercise modes in the Timed Up and Go Test (TUGT) (MD = −2.52, 95% CI: −5.95 to 0.91, *p* = 0.15) or the Functional Ambulation Category scale (FAC) (MD = 0.28, 95% CI: −0.21 to 0.76, *p* = 0.26). Conclusion: Based on the improvement in the Berg Balance Scale, peak oxygen uptake, Fugl–Meyer Assessment, and Functional Independence Measure, we can state that aquatic exercise offers better advantages than land-based exercise for patients’ balance, motor function, cardiorespiratory fitness, and functional independence.

## 1. Introduction

Stroke is a neurological disease caused by the obstruction of normal blood flow due to vessel rupture or blockage, causing damage to brain tissue [1]. It is ranked as the second leading cause of death worldwide, with an annual mortality rate of about 5.5 million. Not only does the burden of stroke lie in its high mortality rate, but its high morbidity also results in up to 50% of survivors being chronically disabled [2]. It is reported that up to 35% of stroke survivors with initial leg paralysis are unable to regain physical function and 20–25% are unable to walk without full physical assistance [3]. In addition, there are several long-term physiological, mental, and psychological problems post-stroke, including movement and function, balance, cognition, and emotional problems [3]. Thus, effective rehabilitation is highly important for stroke patients.

Various therapies are applied in the process of a stroke patient’s rehabilitation, such as psychotherapy [4], physical therapy [5], and exercise therapy [6,7]. Exercise therapies have been used in attempts to prevent physical inactivity and the resultant secondary complications in stroke patients, which has been suggested to be an effective approach [8]. Previous studies showed that LE is an effective intervention method for improving muscle strength, walking capacity, balance, and motor function [9,10]. However, although LE is beneficial for stroke patients, it may cause some negative impacts patients’ joints and muscles. This may result in stress fractures, injuries, and soreness in the muscles, all of which contribute to a reduction in physical activity and fitness [11]. Water is an excellent medium for achieving maximal exercise levels in those with or without disabilities [8]. The environmental characteristics of water influence physiological processes, motor activity, and spasticity, providing the patient with an enabling and motivating environment [12]. AE has also been shown to support normal body weight, which produces less musculoskeletal stress [13]. Buoyancy helps compensate for the gravity present on dry land and is, therefore, highly useful for therapy [14]. When Kim et al. compared the effects of AE and LE using the Berg Balance Scale (BBS), Functional Independence Measure (FIM), and Timed Up and Go Test (TUGT), the AE and LE groups showed significant differences across all pre- and post-experiment variables. However, in the between-group comparison, the AE group was significantly different from the LE group in both BBS and FIM [15]. When Tripp et al. compared the effects of AE and LE using BBS and Functional Ambulation Category scale (FAC), it was evident that the two exercise groups produced similarly effective results [12]. Lee et al. suggested that AE and LE were effective in improving patients’ peak oxygen uptake (VO_2_peak), Fugl–Meyer Assessment (FMA), and BBS compared with the baseline, although there were no significant differences between the two groups [16].

Many studies have shown that the two exercise intervention programs are effective at improving the cardiorespiratory fitness, motor function, balance, and functional independence of stroke patients [10,11,12,13,14,15]. However, due to the limitations of sample size and the differences between research designs and interventions, the results are not comparable and are, therefore, controversial. There have been some meta-analyses of exercise interventions using AE and LE in patients with stroke [17,18]. However, no meta-analysis has exclusively explored the effects of AE and LE on different variables in stroke patients. Thus, this meta-analysis objectively evaluates the effects of AE and LE on the cardiorespiratory fitness, motor function, balance, and functional independence of stroke patients so as to provide a theoretical basis for the exercise rehabilitation of patients with stroke.

## 2. Methods

This meta-analysis was performed in accordance with the PRISMA (Preferred Items for Reporting of Systematic reviews and Meta-Analyses) guidelines [19].

### 2.1. Search Strategy

The electronic databases PubMed, Embase, Cochrane Library, Web of Science, China National Knowledge Infrastructure, VIP Database, and Wanfang Data were searched for this meta-analysis. The results of Randomized Controlled trials (RCTs) were collected between the earliest available date and April 2021 using the following terms: (Aquatic Sport OR Aquatic Exercise OR Aquatic Training OR Water-based Sports OR Water Sport) AND (Stroke OR Cerebrovascular Accident OR Cerebral Stroke OR Brain Vascular Accident OR Apoplexia OR Cerebral Vascular Insufficiency), without any limitations. Meanwhile, the references of articles included in other systematic reviews or meta-analyses were reviewed to identify other possible eligible studies. A detailed summary of the literature search is depicted in Table 1.

### 2.2. Selection Criteria

The inclusion criteria for this meta-analysis were full-text research articles published in peer-reviewed academic journals in Chinese or English language. The exclusion criteria were: (1) non-randomized controlled trial, (2) the outcome does not meet the requirements, (3) there is a significant difference between the baseline values of the two exercise groups (*p* < 0.05).

Two researchers independently screened the studies by reading the titles and abstracts and excluded irrelevant studies. Subsequently, the articles that met the standards were collected and downloaded. Unqualified studies were excluded by reading the full text. Differences in the assessment of study eligibility were resolved by discussion.

### 2.3. Quality Assessment

The methodological quality and risk of bias of the studies included were assessed by two authors using the Cochrane Handbook for Systematic Reviews of Interventions 5.0.1, which includes random sequence generation, allocation concealment, the blinding of participants and personnel, the blinding of outcome assessment, incomplete outcome data, selective reporting, and other biases. The risk of bias was assessed in each domain as “low risk”, “unclear”, or “high risk” [20]. The higher the total score, the higher the methodological quality of the study (5–7 high, 3–4 moderate, 0–2 low). Disagreements were resolved by consensus.

### 2.4. Data Extraction

All data were independently extracted by an investigator and checked for accuracy by another reviewer. Collected data included authors’ names, year of publication, country in which the study was conducted, characteristics of the participants (sample size, gender and age), intervention description (exercise mode, duration, water temperature/depth and exercise program), outcomes, and quality assessment score.

### 2.5. Statistical Analysis

Statistical analyses were performed using Review Manager 5.3 (Nordic Cochrane Centre, Copenhagen, Denmark) and Stata MP 15.0 (StataCorp, Pyrmont, Australia). Effect sizes for continuous variables were expressed as mean difference (MD), each with 95% confidence intervals (95% CI). Heterogeneity among studies was examined with Cochran’s Q and I^2^ statistics, in which values greater than 50% indicated significant heterogeneity; a random-effects model was chosen [21]. Overall effects were considered significant when *p* < 0.05. Sensitivity analysis with the exclusion of each study was conducted to investigate the possible effects on heterogeneity and the overall effects. Finally, Egger’s regression model was used to assess publication bias.

## 3. Results

### 3.1. Study Selection

**Figure 1:**
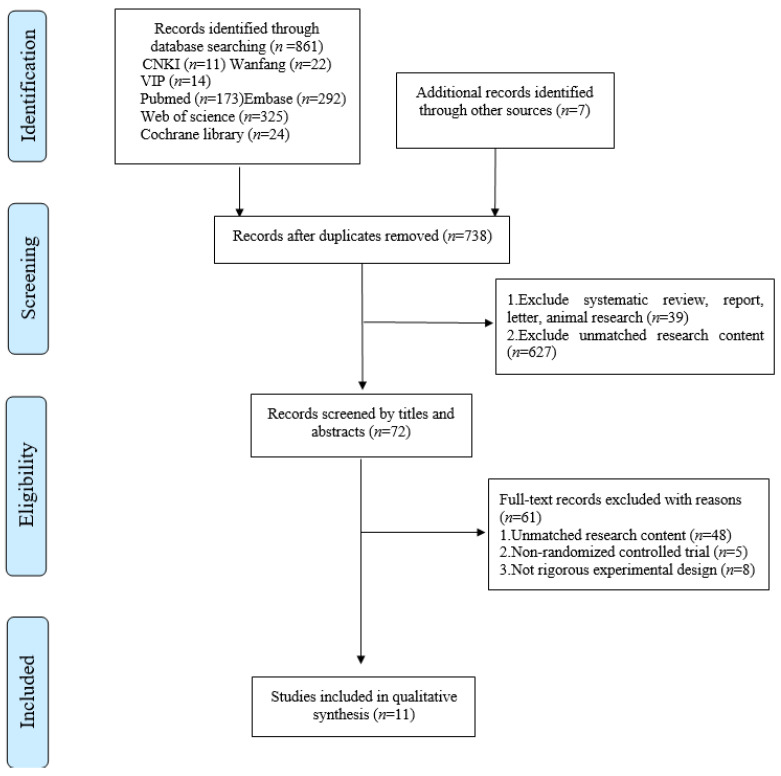
Flow diagram of literature selection.

The initial research resulted in 868 references. After duplicates were removed, the titles and abstracts of 738 studies were reviewed. Following a screening of potential studies, 666 studies were excluded and 72 studies were retrieved in full-text, 61 of which did not match the eligibility criteria. The final 11 studies were included in the meta-analysis (Figure 1).

### 3.2. Risk of Bias Assessment

**Figure 2:**
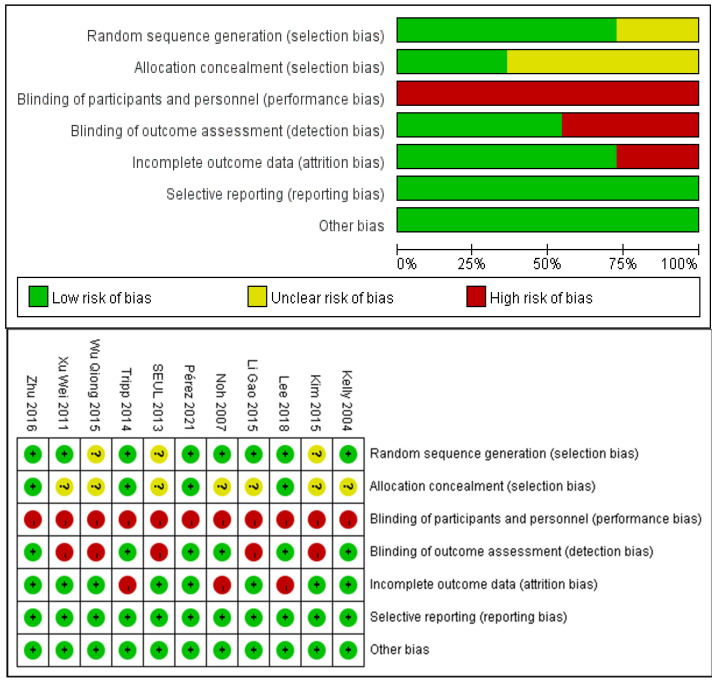
Analysis of the risk of bias in accordance with the Cochrane collaboration guideline.

The risk of bias for included studies was evaluated with the Cochrane Risk of Bias Tool and the results are shown in Figure 2. Eight studies described a random sequence generation and were evaluated as low risk. Four studies demonstrated a low risk of bias through allocation concealment using a sealed envelope. Six studies were assessor-blinded and were classified as at low risk of detection bias. Three studies demonstrated a high risk of bias through incomplete outcome data. There was no selective reporting and no other bias in the included studies.

### 3.3. Study Characteristics

The characteristics of the included studies are shown in Table 2. The eleven included studies involved a total of 369 patients (187 AE and 182 LE) with stroke. Among these studies, four were conducted in Korea; four were conducted in China; and three were separately conducted in Canada, Germany, and Spain. The age of the participants varied from forty to eighty years. Intervention duration ranged from two to twelve weeks, with a frequency of exercise training ranging from two to six days per week. Nine studies reported the water temperature and ten studies reported the water depth.

## 4. Meta-Analysis

### 4.1. Berg Balance Scale (BBS)

**Figure 3:**
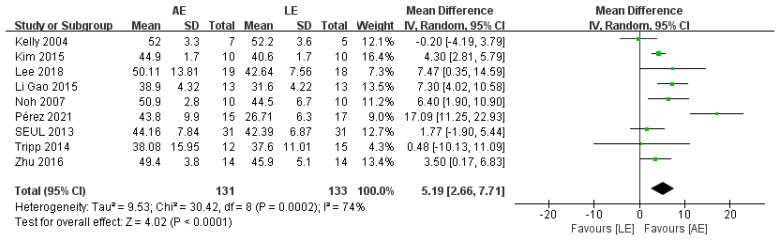
Forest plot: effects of BBS.

BBS was reported by nine studies, including 264 patients with stroke. The meta-analysis showed a significant improvement for patients in the AE group compared with the LE group (random-effects model: MD = 5.19; 95% CI: 2.66 to 7.71; *p* < 0.0001) (Figure 3). The test for heterogeneity was significant (*p* = 0.0002; I^2^ = 74%). Subgroup analyses based on intervention duration and exercise frequency were performed to explore heterogeneity. The results of the subgroup analyses showed that intervention duration and exercise frequency were not potential factors that lead to heterogeneity. Sensitivity analysis did not change the statistical significance of the overall results. The exclusion of the study conducted by Perez [26], which provided inferior evidence for the effect of AE on BBS, significantly improved the homogeneity. The heterogeneity may have been caused by the long intervention duration (12 weeks).

### 4.2. Fugl–Meyer Assessment (FMA)

**Figure 4:**
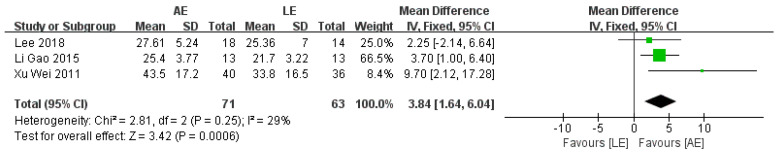
Forest plot: effects of FMA.

FMA was reported by three studies that included a total of 134 patients with stroke. The meta-analysis showed a significant improvement for patients in the AE group compared with the LE group (fixed-effects model: MD = 3.84; 95% CI: 1.64 to 6.04; *p* = 0.0006) (Figure 4). The test for heterogeneity was not significant (*p* = 0.25; I^2^ = 29%).

### 4.3. Timed Up and Go Test (TUGT)

**Figure 5:**
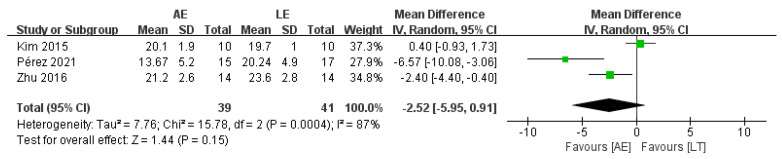
Forest plot: effects of TUGT.

Three studies with a total of 80 patients reported no difference in TUGT between AE and LE (random-effects model: MD = −2.52; 95% CI: −5.95 to 0.91; *p* = 0.15) (Figure 5). The test for heterogeneity was significant (*p* = 0.0004; I^2^ = 87%). Sensitivity analyses were conducted to explore potential sources of heterogeneity; the exclusion of individual studies did not substantially alter the heterogeneity.

### 4.4. Functional Ambulation Category Scale (FAC)

**Figure 6:**
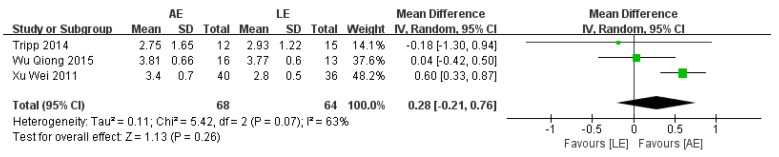
Forest plot: effects of FAC.

FAC was reported by three studies that included a total of 132 participants with stroke. The meta-analysis showed no significance for participants in the AE group compared with the LE group (random-effects model: MD = 0.28; 95% CI: −0.21 to 0.76; *p* = 0.26) (Figure 6). The test for heterogeneity was significant (*p* =0.07; I^2^ = 63%). Sensitivity analyses were conducted to explore potential sources of heterogeneity; the exclusion of individual studies did not substantially alter the heterogeneity.

### 4.5. Peak Oxygen Uptake (VO_2_peak)

**Figure 7:**
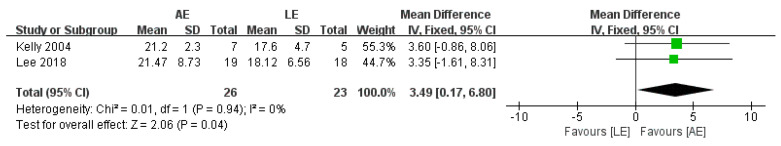
Forest plot: effects of VO_2_peak.

VO_2_peak was reported by two studies, including 49 participants with stroke. The aggregate results of these studies showed that AE was associated with a significantly improved VO_2_peak (fixed-effects model: MD = 3.49; 95% CI: 0.17 to 6.8; *p* = 0.04) (Figure 7). The test for heterogeneity was not significant (*p* = 0.04; I^2^ = 0%).

### 4.6. Functional Independence Measure (FIM)

**Figure 8:**
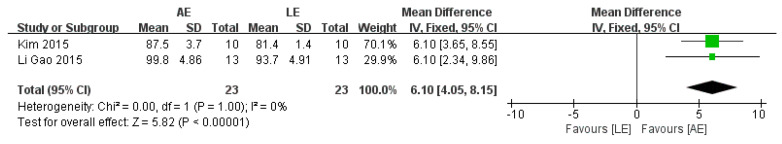
Forest plot: effects of FIM.

Three studies with a total of 46 patients reported a significant difference in FIM between AE and LE (fixed-effects model: MD = 6.1; 95% CI: 4.05 to 8.15; *p* < 0.00001) (Figure 8). The test for heterogeneity was not significant (*p* = 1.00; I^2^ = 0%).

### 4.7. Publication Bias

**Figure 9:**
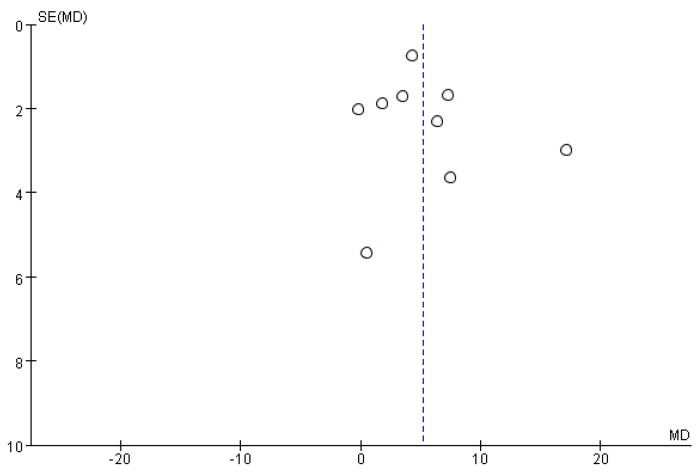
Funnel plot for BBS from the included studies.

Funnel plots did not show any significant publication bias for the primary outcome of BBS, meaning that there was no asymmetric relationship between treatment effects and study size (Figure 9).

## 5. Discussion

Exercise therapy is effective as a rehabilitation tool for improving functional recovery and promoting neural plasticity [27]. However, there is still controversy regarding the mode that can yield optimal beneficial effects in stroke patients. Previous meta-analyses involved the exercise intervention of AE and LE in patients with stroke [17,18]; however, there were some disputes about the intervention programs. The above two meta-analyses included some studies with AE combined with LE vs. LE, so it is unclear whether the final significance is caused by combined exercise (AE combined with LE) or single AE. This study was the first to explore the effect of single aquatic exercise and single land-based exercise on different variables in stroke patients, especially cardiorespiratory fitness. The results of this meta-analysis showed that based on the improvement of Berg Balance Scale, peak oxygen uptake, Fugl–Meyer Assessment, and Functional Independence Measure, AE offers better advantages than LE for patients’ balance, motor function, cardiorespiratory fitness, and functional independence.

Balance impairment is one of the major physical problems for patients with stroke and leads to limitation in the performance of daily living activities and participation in society [28]. The Berg Balance Scale (BBS), which has been shown to be a valid and reliable measure, consists of 14 tasks that challenge balance while the subject is sitting, standing, or stepping (minimum score = 0 and maximum score = 56, where higher scores indicate better balance) [29]. Patients with a BBS score of less than 45 are known to have an increased risk of falling [30]. Thus, this meta-analysis took BBS as the primary outcome to measure postural stability. The results of this meta-analysis suggested that AE improved BBS in patients with stroke significantly more than LE (MD = 5.19, 95% CI: 2.66 to 7.71, *p* < 0.0001). The improvement of postural stability is described as the most important prognostic factor in stroke patients for achieving independent gait ability [31]. The mechanism of AE improving BBS may be explained by the following aspects: (1) Because of the effect of water resistance, the speed of water-based exercise is usually slower than land-based exercise. As a result, the weight bearing time of the lower extremities increases, which helps to improve the muscle strength of the lower extremities of the affected side [24]. (2) The patient’s exercise in the water is affected by the buoyancy of water against gravity. Under the support of buoyancy and hydrostatic pressure, the patients cannot fall easily and can move freely, which may lead to the improvement of posture, balance, and coordination ability [32].

The Fugl–Meyer Assessment (FMA) is a stroke-specific, performance-based impairment index that assesses motor function, balance, sensation, joint function, and pain in patients with post-stroke hemiplegia. It comprises 155 items, and each item is rated on a three-point ordinal scale (0, cannot perform; 1, can perform partially; 2, can perform fully) [16]. The meta-analysis suggested that there was a significant difference in FMA between AE and LE (fixed-effects model: MD = 3.84; 95% CI: 1.64 to 6.04; *p* = 0.0006). The Functional Independence Measure (FIM) consists of 13 items related to mobility and 5 related to recognition. The items are scored on a scale of 1–7, with 126 possible total points; higher scores indicate better independence [15]. The meta-analysis suggested that there was a significant difference in FIM between AE and LE (fixed-effects model: MD = 6.1; 95% CI: 4.05 to 8.15; *p* < 0.00001). A possible explanation for the improvements in FMA and FIM is that the distribution of body gravity tends to be symmetrical when patients exercise in water. The buoyancy of water can partly make up for the lack of strength of the hip flexion muscle group on the affected side and reduce pathological hip abduction and external rotation, finally improving gait symmetry [33].

Cardiovascular disease is the leading prospective cause of death in people with stroke [34]. VO_2_peak is considered to be the best predictor of survival in cardiovascular diseases [35]. The meta-analysis showed that AE improved the VO_2_peak of 3.49 mL/kg.min in patients with stroke more significantly than LE. Compared with LE, the higher intensity and greater resistance of AE may result in an increase in plasma volume and erythrocyte volume. Meanwhile, exercise in water can also increase central blood volume and vital capacity, as well as improving cardiopulmonary function [33].

Functional Ambulation Categories (FAC) describe the dependency on assistance for gait on a five-point scale. The meta-analysis results showed that there was no significant difference in FAC between the two exercise programs (random-effects model: MD = 0.28; 95% CI: −0.21 to 0.76; *p* = 0.26). TUGT was created and validated to assess dynamic balance, mobility and the risk of falls in older persons [36]. The meta-analysis suggested that there was no significant difference in TUGT between AE and LE (random-effects model: MD = −2.52; 95% CI: −5.95 to 0.91; *p* = 0.15). These outcomes still need to be further elucidated in large and well-designed studies.

There are some limitations to this meta-analysis: (1) There is significant heterogeneity with respect to the outcome of BBS. Although various subgroups (i.e., exercise duration, exercise frequency) were assessed to explore heterogeneity, unwanted heterogeneity was still obvious; (2) Some outcomes are in small sample sizes, which may affect the stability of the results. It is hoped that more well-designed studies will further expand these meta-analysis results in the future.

## 6. Conclusions

Based on the improvement of Berg Balance Scale, peak oxygen uptake, Fugl–Meyer Assessment and Functional Independence Measure, aquatic exercise offers better advantages than land-based exercise for patients’ balance, motor function, cardiorespiratory fitness, and functional independence.

## Figures and Tables

**Table 1:** Search strategy on PubMed.

#1	Search “Stroke”[Mesh]
#2	Search (((((((((((((Strokes [Title/Abstract]) OR (Cerebrovascular Accident [Title/Abstract])) OR (CVA (Cerebrovascular Accident) [Title/Abstract])) OR (Cerebrovascular Apoplexy [Title/Abstract])) OR (Vascular Accident, Brain [Title/Abstract])) OR (Brain Vascular Accident [Title/Abstract])) OR (Cerebrovascular Stroke [Title/Abstract])) OR (Stroke, Cerebrovascular [Title/Abstract])) OR (Cerebral Stroke [Title/Abstract])) OR (Stroke, Cerebral [Title/Abstract])) OR (Stroke, Acute [Title/Abstract])) OR (Acute Stroke [Title/Abstract])) OR (Cerebrovascular Accident, Acute [Title/Abstract])) OR (Acute Cerebrovascular Accident [Title/Abstract])
#3	Search #1 OR #2
#4	Search ((aquatic [Title/Abstract]) OR (aquatic therapy [Title/Abstract])) OR (aquatic exercise [Title/Abstract])
#5	Search #3 AND #4

**Table 2:** Characteristics of the studies included in the meta-analysis.

Study	Country	Characteristics of Patients			Intervention					Outcomes	Quality Assessment
		Sample size (AE/LE)	Gender (M/F)	Age (Years) (MEAN ± SD)	Duration	Water Temperature	Depth	Exercise Program (AE/LE)			
Kelly, 2004 [22]	Canada	12 (7/5)	AE: 7 (6/1) LE: 5 (5/0)	AE: 61.9 ± 9.4 LE: 63.4 ± 8.4	8 weeks 3/wks 60 min	26–28 °C	chest- level	Aerobic training: ① 10 min land-based stretching ② 5 min warm-up in the water ③ 30 min moderate to high aerobic activities ④ 5 min cool down ⑤ 10 min stretching inthe water	Strength training: ① 5 min warm-up ② 42 min upper-extremity strengthening ③ 5 min cool-down	①⑤	5
Noh, 2007 [8]	Korea	25 (13/12)	AE: 13 (7/6) LE: 12 (4/8)	AE:61.9 ± 10.1 LE:66 ± 11.4	8 weeks 3/weeks 60 min	34 °C	115 cm	Halliwick and Ai Chi training: ① 10 min warm-up ② 20 min Halliwick method ③ 20 min rounding and balancing according to the Ai Chi method ④ 10 min cool-down	Strength and balance training: ① 10 min warm-up ② 40 min lower extremity strengthening, upper-extremity strengthening and gait training ③ 10 min cool-down	①	4
Xu Wei, 2011 [23]	China	76 (40/36)	AE: 40 (23/17) LE:36 (20/16)	AE: 51.3 ± 8.2 LE: 49.3 ± 7.4	4 weeks 6/weeks 30 min	/	130–140 cm	Aerobic and balance training: ① Warm-up ② Hemiplegic gymnastics ③ Water-based walking	Aerobic training: Land-based treadmill walking	②④	4
SEUL, 2013 [6]	Korea	62 (31/31)	AE: 31 (15/16) LE:31 (13/18)	AE: 56.1 ± 7.3 LE: 56.6 ± 10	6 weeks 3/weeks 40 min	33.5 °C	110 cm	Strength training: ① 5 min warm-up ② 30 min main exercises (one-legged knee flexion, toe stand, one-legged stance, knee flexion of both legs, eeight shift) ③ 5 min cool-down	Strength training: ① 5 min warm-up ② 30 min main exercises (one-legged knee flexion, toe stand, one-legged stance, knee flexion of both legs, weight shift) ③ 5 min cool-down	①	3
Tripp, 2014 [16]	Germany	30 (14/16)	AE: 14 (9/5) LE: 16 (10/6)	AE: 64.8 ± 15 LE: 65 ± 15.1	2 weeks 5/weeks 45 min	/	/	Halliwick training: ① 5 min warm-up ② 5 min were for exercises in water familiarization and mental adaption ③ 15 min for exercising rotational control ④ 15 min locomotion under various disturbances and in changing water depths ⑤ 5 min cool-down	No standard intervention programs: An individual mix of different treatment concepts, task-specific exercising of various tasks in the area of mobility and possibly treadmill training	①④	5
Kim, 2015 [15]	Korea	20 (10/10)	AE: 10 (5/5) LE: 10 (5/5)	AE: 69.1 ± 3.2 LE: 68 ± 3.1	6 weeks 5/weeks 30 min	31–33 °C	110 cm	Proprioceptive training: Proprioceptive neuromuscular facilitation lower extremity patterns in water	Proprioceptive training: Proprioceptive neuromuscular facilitation lower extremity patterns on the ground	①③⑥	3
Wu Qiong, 2015 [24]	China	29 (16/13)	AE: 16 (10/6) LE: 13 (9/4)	AE: 50.94 ± 11.06 LE: 51.38 ± 10.62	4 weeks 6/weeks 20 min	37 °C	xiphoid level	Aerobic training: Underwater treadmill training	Aerobic training: Land-based treadmill training	④	3
Li Gao, 2015 [25]	China	26 (13/13)	AE: 13 (8/5) LE: 13 (8/5)	AE: 54.6 ± 5.58 LE: 55.4 ± 5.62	9 weeks 5/weeks 45 min	37 °C	120- 150 cm	Strength and aerobic training: Underwater walking and strengthening exercises	Strength and aerobic training: Land-based walking and strengthening exercises	①②⑥	4
Zhu, Z 2016 [7]	China	28 (14/14)	AE: 14 (12/2) LE: 14 (10/4)	AE: 56.6 ± 6.9 LE: 57.1 ± 8.6	4 weeks 5/weeks 45 min	34–36 °C	140 cm	Strength and aerobic training: ① 5 min warm-up ② 30 min strengthening exercises and treadmill exercises ③ 10 min cool-down	Strength and aerobic training: ① 5 min warm-up ② 30 min strengthening exercises and treadmill exercises ③ 10 min cool-down	①③	6
Lee, S. Y, 2018 [16]	Korea	37 (19/18)	AE: 19 (9/10) LE: 18 (10/8)	AE: 57.58 ± 13.98 LE: 63.67 ± 11.37	4 weeks 5/weeks 30 min	30–33 °C	popliteal level	Aerobic training: ① 5 min warm-up ② 20 min water-based running ③ 5 min cool-down	Aerobic training: 30 min land-based aerobic exercise	①②⑤	5
Pérez, 2021 [26]	Spain	32 (15/17)	AE: 15 (7/8) LE: 17 (8/9)	AE: 63.8 ± 13.6 LE: 62.7 ± 13.4	12 weeks 2/weeks 45 min	30 °C	110 cm	Aerobic training: ① 10 min warm-up ② 30 min Ai Chi program ③ 5 min cool-down	Strength and aerobic training: ① 10 min warm-up ② 30–40 min strength training and aerobic exercises ③ 5 min cool-down	①③	6

Abbreviations: AE: aquatic exercise; LE: land-based exercise; M/F: male/female. Outcomes: ① BBS; ② FMA; ③ TUGT; ④ FAC; ⑤ VO_2_ peak; ⑥ FIM.

## Data Availability

The data that support the findings of the study are available from the corresponding author, upon reasonable request.

## References

[1] De La Cruz S.P. (2020). Influence of an Aquatic Therapy Program on Perceived Pain, Stress, and Quality of Life in Chronic Stroke Patients: A Randomized Trial. Int. J. Environ. Res. Public Health.

[2] Donkor E.S. (2018). Stroke in the 21st Century: A Snapshot of the Burden, Epidemiology, and Quality of Life. Stroke Res. Treat..

[3] Han P., Zhang W., Kang L., Ma Y., Fu L., Jia L., Yu H., Chen X., Hou L., Wang L. (2017). Clinical Evidence of Exercise Benefits for Stroke. Adv. Exp. Med. Biol..

[4] Lincoln N., Flannaghan T. (2003). Cognitive Behavioral Psychotherapy for Depression Following Stroke. Stroke.

[5] Khan F., Rathore C., Kate M., Joy J., Zachariah G., Vincent P.C., Varma R.P., Radhakrishnan K. (2019). The comparative efficacy of theta burst stimulation or functional electrical stimulation when combined with physical therapy after stroke: A randomized controlled trial. Clin. Rehabil..

[6] Han S.K., Kim M.C., An C.S. (2013). Comparison of Effects of a Proprioceptive Exercise Program in Water and on Land the Balance of Chronic Stroke Patients. J. Phys. Ther. Sci..

[7] Zhu Z., Cui L., Yin M., Yu Y., Zhou X., Wang H., Yan H. (2015). Hydrotherapy vs. conventional land-based exercise for improving walking and balance after stroke: A randomized controlled trial. Clin. Rehabil..

[8] Noh D.K., Lim J.-Y., Shin H.-I., Paik N.-J. (2008). The effect of aquatic therapy on postural balance and muscle strength in stroke survivors—A randomized controlled pilot trial. Clin. Rehabil..

[9] Lee J., Stone A.J. (2019). Combined Aerobic and Resistance Training for Cardiorespiratory Fitness, Muscle Strength, and Walking Capacity after Stroke: A Systematic Review and Meta-Analysis. J. Stroke Cerebrovasc. Dis..

[10] Kim H., Lee H., Seo K. (2013). The Effects of Dual-Motor Task Training on the Gait Ability of Chronic Stroke Patients. J. Phys. Ther. Sci..

[11] Saleh M.S.M., Rehab N.I., Aly S.M.A. (2019). Effect of aquatic versus land motor dual task training on balance and gait of patients with chronic stroke: A randomized controlled trial. NeuroRehabilitation.

[12] Tripp F., Krakow K. (2014). Effects of an aquatic therapy approach (Halliwick-Therapy) on functional mobility in subacute stroke patients: A randomized controlled trial. Clin. Rehabil..

[13] Tamin T.Z., Loekito N. (2018). Aquatic versus land-based exercise for cardiorespiratory endurance and quality of life in obese patients with knee osteoarthritis: A randomized controlled trial. Med. J. Indones..

[14] De La Cruz S.P. (2020). Comparison of Aquatic Therapy vs. Dry Land Therapy to Improve Mobility of Chronic Stroke Patients. Int. J. Environ. Res. Public Health.

[15] Kim E.-K., Lee D.-K., Kim Y.-M. (2015). Effects of aquatic PNF lower extremity patterns on balance and ADL of stroke patients. J. Phys. Ther. Sci..

[16] Lee S.Y., Im S.H., Kim B.R., Han E.Y. (2018). The Effects of a Motorized Aquatic Treadmill Exercise Program on Muscle Strength, Cardiorespiratory Fitness, and Clinical Function in Subacute Stroke Patients. Am. J. Phys. Med. Rehabil..

[17] Nayak P., Mahmood A., Natarajan M., Hombali A., Prashanth C., Solomon J.M. (2020). Effect of aquatic therapy on balance and gait in stroke survivors: A systematic review and meta-analysis. Complement. Ther. Clin. Pr..

[18] Giuriati S., Servadio A., Temperoni G., Curcio A., Valente D., Galeoto G. (2021). The effect of aquatic physical therapy in patients with stroke: A systematic review and meta-analysis. Top. Stroke Rehabil..

[19] Moher D., Shamseer L., Clarke M., Ghersi D., Liberati A., Petticrew M., Shekelle P., Stewart L.A., PRISMA-P Group (2015). Preferred reporting items for systematic review and meta-analysis protocols (PRISMA-P) 2015 statement. Syst. Rev..

[20] Higgins J.P.T., Green S. (2011). Cochrane Handbook for Systematic Reviews of Interventions Version 5.1.0. Cochrane Collab..

[21] Higgins J.P.T., Thompson S.G., Deeks J.J., Altman D.G. (2003). Measuring inconsistency in meta-analyses. BMJ.

[22] Chu K.S., Eng J.J., Dawson A.S., Harris J.E., Ozkaplan A., Gylfadóttir S. (2004). Water-based exercise for cardiovascular fitness in people with chronic stroke: A randomized controlled trial. Arch. Phys. Med. Rehabil..

[23] Xu W., Fan J.T., Zhang L.Y., Yang X., Wang Y.Z. (2011). Effects of water sports training and weight-loss walking training on walking ability of stroke patients with hemiplegia. Chin. J. Phys. Med. Rehabil..

[24] Wu Q., Cong F., Song G. (2015). Comparison of Effects between Underwater and Body Weight Support Treadmill Training on Walking and Balance in Hemiplegics after Stroke. Chin. J. Rehabil. Theory Pract..

[25] Li G. (2015). Effect of muscle strength training combined with water walking training on lower limb function recovery of stroke patients with hemiplegia. Chin. J. Phys. Med. Rehabil..

[26] De La Cruz S.P. (2021). Comparison between Three Therapeutic Options for the Treatment of Balance and Gait in Stroke: A Randomized Controlled Trial. Int. J. Environ. Res. Public Health.

[27] Pin-Barre C., Laurin J. (2015). Physical Exercise as a Diagnostic, Rehabilitation, and Preventive Tool: Influence on Neuroplasticity and Motor Recovery after Stroke. Neural Plast..

[28] Ijmker T., Houdijk H., Lamoth C., Jarbandhan A.V., Rijntjes D., Beek P.J., van der Woude L. (2013). Effect of Balance Support on the Energy Cost of Walking After Stroke. Arch. Phys. Med. Rehabil..

[29] Berg K.O., Maki B.E., Williams J.I., Holliday P.J., Wood-Dauphinee S.L. (1992). Clinical and laboratory measures of postural balance in an elderly population. Arch. Phys. Med. Rehabil..

[30] Andersson A.G., Kamwendo K., Seiger Å., Appelros P. (2006). How to identify potential fallers in a stroke unit: Validity indexes of four test methods. J. Rehabil. Med..

[31] Kollen B., Van De Port I., Lindeman E., Twisk J., Kwakkel G. (2005). Predicting Improvement in Gait After Stroke. Stroke.

[32] Li W., Chaoqin D. (2014). The Effect of Water Intensive Walking Training on the Recovery of Walking Ability of Hemiplegia Patients with Stroke. Chin. J. Rehabil. Med..

[33] Wang J., Huang B., Yang Z. (2015). Effect of treadmill training in water on walking ability of stroke patients. Chin. J. Rehabil. Med..

[34] Roth E.J. (1993). Heart disease in patients with stroke: Incidence, impact, and implications for rehabilitation part 1: Classification and prevalence. Arch. Phys. Med. Rehabil..

[35] Opasich C., Pinna G.D., Bobbio M., Sisti M., Demichelis B., Febo O., Forni G., Riccardi R., Riccardi P., Capomolla S. (1998). Peak exercise oxygen consumption in chronic heart failure: Toward efficient use in the individual patient. J. Am. Coll. Cardiol..

[36] Podsiadlo D., Richardson S. (1991). The TiMed “Up & Go”: A Test of Basic Functional Mobility for Frail Elderly Persons. J. Am. Geriatr. Soc..

